# Coping mechanisms used by the families of mental health care users in Mahikeng sub-district, North West province

**DOI:** 10.4102/hsag.v26i0.1586

**Published:** 2021-08-16

**Authors:** Tshepang P. Modise, Isaac O. Mokgaola, Leepile A. Sehularo

**Affiliations:** 1 School of Nursing Science, Faculty of Health Sciences, North-West University, Mmabatho, South Africa

**Keywords:** coping mechanisms, families, health care users, mental disorders, challenges of families of mental health care users

## Abstract

**Background:**

Families of the mental health care users (MHCUs) face different challenges in dealing with, supporting and caring for MHCUs on a daily basis. The divergent coping mechanisms that the family members use aim to lower the negative, psychological and emotional impact of the stress. These include: escape, avoidance and denial.

**Aim:**

To explore, describe and contextualise coping mechanisms used by the families of MHCUs and to suggest recommendations for improving their coping mechanisms in Mahikeng sub-district, North West province (NWP), South Africa.

**Setting:**

The study was conducted in three community health centres in Mahikeng sub-district, NWP, South Africa.

**Methods:**

A qualitative-exploratory-descriptive and contextual research design was used. Non-probability convenience and purposive sampling techniques were used to select participants. WhatsApp video calls were used to collect data which were analysed following Creswell’s six steps of qualitative data analysis.

**Results:**

The study established three themes namely; challenges experienced by the family members, coping mechanism used by the family members, and suggestions for improvement in the coping mechanisms for the family members.

**Conclusion:**

The findings of this study show that the family members of MHCUs are faced with different challenges. Some of the coping mechanisms used by the family members are insufficient and require improvement to enable them to cope effectively. When the coping mechanisms of the family members of MHCUs are improved, their well-being and that of the MHCUs might improve significantly.

**Contribution:**

The findings of this study provides information that may be used to improve the coping mechanisms of the families of MHCUs in the NWP, South Africa.

## Introduction

Mental disorders affect both the mental health care users (MHCUs) and their family members (Tristiana et al. 2018:1). According to a qualitative study conducted by Spaniol and Nelson (2015:765), in Switzerland, families of the MHCUs often become physically and emotionally exhausted by the additional burden of caring for their loved ones. Some of their responsibilities (burdens) include: seeking mental healthcare services and support for their mentally ill family members, educating themselves about mental illnesses, being mindful of the needs of other family members, and finding time to care for themselves. In a study that was conducted in Columbia, Hartney and Barnard (2015:355) argue that long term continuous care giving leads to significant stress often referred to as the ‘family burden’ or the ‘care-giving burden’. Mental health problems such as schizophrenia, mood or substance use disorders exert a toll on the families of the MHCUs who are negatively affected both economically as well as emotionally.

In spite of the above concerns, literature shows that different families use different coping mechanisms when caring for their MHCUs (Ata & Doğan 2018:113; Ong, Ibrahim & Wahab 2016:212; Parks et al. 2018:242). For instance, Ong et al. (2016:212) identify coping mechanisms such as reinterpretation, positive life growth, social support, usage of religion or spirituality, active coping, acceptance, and positive reframing as positively associated with lower distress. On the other hand, coping mechanisms such as self-blame, avoidance, and mental disengagement are positively associated with higher distress (Ong et al. 2016:212). In their quantitative study conducted in Turkey, Ata and Doğan (2018:113) state that families of the MHCUs use coping mechanisms such as crying, denial, fury, withdrawal from social life, aggressive behaviours, positive thinking, getting information, getting support from family and neighbours, and seeking social support. Researchers such as Parks et al. (2018:242) argue that families use certain approach-oriented coping mechanisms such as seeking information and positive communication. For instance, families making efforts to understand mental illness that their MHCUs had were correlated with a higher level of education among families of the MHCUs, while avoidant coping mechanisms were more common among those with a lower education level and whose MHCU had a severe mental illness.

Based on the above information as well as the researcher’s personal experience as a professional nurse for almost 5 years, it is clear that the families of the MHCUs face different challenges in dealing with, supporting and caring for the MHCUs on a daily basis, particularly in Mahikeng sub-district, North West province (NWP). This requires family members to use various coping mechanisms, some adaptive and some maladaptive in nature. The different coping mechanisms that the family members use aim to minimise the negative emotional impact of the stressor. These include: escape, avoidance, denial or looking to religion to deal with behavioural problems of the MHCUs. Consequently, this makes dealing with, supporting and caring for them easier (Madathumkovilakath et al. 2018:29). From the reviewed literature, it seems studies focusing on the coping mechanisms employed by the families of the MHCUs in the NWP are scarce. In response to the above concerns, this study aimed at exploring, describing and contextualising coping mechanisms used by the families of the MHCUs with the view to recommending strategies for improving their coping mechanisms in Mahikeng sub-district, NWP.

## Research methodology

### Research design

A qualitative-exploratory-descriptive and contextual research design was used to explore, describe and contextualise coping mechanisms used by the families of the MHCUs in Mahikeng sub-district, NWP. WhatsApp video calls were used to collect data, which was analysed following Creswell’s six steps of qualitative data analysis.

### Study setting

The study was conducted in three community health centres in Mahikeng sub-district, NWP. There are four districts in the province namely, Ngaka Modiri-Molema, Dr Kenneth Kaunda, Dr Ruth Segomotsi Mompati and Bojanala. There are approximately 19 sub-districts in the province namely, Mahikeng, Ditsobotla, Tswaing, Rustenburg, Ramotsere Moiloa, Tlokwe, Naledi, Ventersdorp, Matlosana, Greater Taung, Moses Kotane, Moretele, Madibeng, Lekwa Teemane, Mamusa, Kagisano-Molopo, Maquassi Hills, Kgetlengriver and Ratlou sub-district. The researcher chose Mahikeng sub-district as it was noticed that the families of the MHCUs in this district are severely affected by the challenges of dealing with supporting and caring for the MHCUs on a daily basis. Based on these concerns, the researcher became interested in understanding how the families of the MHCUs cope. The three clinics where the study was conducted were selected with the help of the sub-district manager.

### Population and sampling

The population of the study included all family members of MHCUs who were over the age of 18 years and residing in Mahikeng sub-district, NWP. A non-probability sampling approach was used. Only those members of the population who were knowledgeable about the topic were selected by an independent person. Convenience and purposive sampling techniques were used to select 10 participants. Data saturation determined sampling size of the study which was 10 participants.

### Data collection method

As a result of the coronavirus 2019 (COVID-19) outbreak, physical contact with participants was avoided to protect them and the researcher from the risk of exposure to COVID-19. WhatsApp video calls were therefore used by the researcher to collect data from the participants. The interviews were free flowing in structure, limited only by the focus of the research. The video call interviews were conducted in a conversational style, albeit with a specific purpose in mind, which was to explore, describe and contextualise coping mechanisms used by families of MHCUs in Mahikeng sub-district, NWP. Communication techniques such as clarifying, summarising and reflection were used by the researcher in facilitating the discussion in all the semi-structured individual interviews. The following open-ended questions were asked in all the semi-structured individual interviews: What are some of the challenges that you experience in caring for your family member who suffers from a mental illness? How do you cope? What helps you to cope? What do you think might help you or other people to cope effectively?

### Data analysis

Data were analysed independently by both the researcher and an independent co-coder following Creswell’s six steps of qualitative data analysis (Creswell 2014:247). The researcher prepared and organised the data through verbatim transcription of all the WhatsApp video call interviews. The researcher and an independent co-coder read the transcribed data from all the WhatsApp video call interviews to get the general tone, and the sense of the data. Coding of all the data was performed. Data were organised by bracketing chunks (or text or image segments) representing a category. The coding process was used to generate a detailed description of categories or themes. Both the researcher and an independent co-coder met to discuss and finalise themes and sub-themes of the study. Subsequently, the researcher presented the themes and sub-themes in a narrative format and lastly, interpreted the final themes and sub-themes.

### Trustworthiness

The following four criteria were followed to ensure trustworthiness of this study: credibility, confirmability, transferability, and dependability as outlined by Taylor, Bogdan and DeVault (2015:50). Credibility was maintained through prolonged engagement with participants before and during data collection. For example, data were collected until saturation was reached. Confirmability was achieved through collection of data using WhatsApp video call interviews, asking open-ended questions as indicated in the data collection process above, ensuring that participants were given enough time and opportunity to express their views without being led by the researcher. Dependability was achieved through a detailed description of the research methodology of the study and verbatim transcription of data, coding, formulation of themes and categories during data analysis. Literature control was done to support the findings of the study. The findings of this qualitative study are not transferable to other settings, but they can be applied. Purposive and convenient sampling techniques and thick description of the research methodology and results also assured transferability of the study.

### Ethical considerations

Ethical clearance was sought and obtained from the North-West University Health Research Ethics Committee (NWU-HREC, Ref: NWU-00959-19-S1). Permission was obtained from the North West Provincial Department of Health (DOH) Ethics Committee, Mahikeng sub-district office as well as operational managers of the clinics where data were collected. Participants were informed by an independent person about their right to choose to participate and the right to withdraw from the study at any given time without being penalised. The researcher ensured the protection of information obtained from the participants in line with *Protection of Personal Information Act* (POPIA) No. 04 of 2013. The researcher ensured that the identity of the participants was protected and not disclosed to anyone outside the research team. This was achieved by assigning pseudonyms to participants during data collection. Participants were only identified as Participant, A, B, or C during data collection.

## Results

WhatsApp video call interviews were conducted with 10 participants, all females. Six (6) of the participants were mothers, three were sisters and one was the daughter of MHCUs. Age of the participants ranged between 27 and 63 years. Table 1 shows the demographic characteristics of the participants.

**TABLE 1:** Demographic information of participants.

Participant number	Age (years)	Gender	Relationship	Socio-economic status	Years looking after MCHU
Participant A	57	Female	Mother	Unemployed	26
Participant B	63	Female	Mother	Pensioner	28
Participant C	38	Female	Sister	Employed	10
Participant D	55	Female	Mother	Unemployed	15
Participant E	27	Female	Daughter	Student	6
Participant F	50	Female	Mother	Domestic worker	16
Participant G	56	Female	Mother	Employed	12
Participant H	49	Female	Mother	Unemployed	17
Participant I	36	Female	Sister	General worker	8
Participant J	42	Female	Sister	Unemployed	17

MHCUs, mental health care users.

### Themes

The following three themes emerged from the findings of the study: challenges experienced by family members, coping mechanisms used by family members as well as suggestions to improve family members’ coping mechanisms (Table 2).

**TABLE 2:** Themes and sub-themes.

Theme	Sub-theme
1. Challenges experienced by family members	Difficult, uncooperative behaviour by MHCUs MHCU substance use Defaulting treatment Stigma because of mental illness Negative attitudes from the nursing staff Insufficient income to care for MHCUs
2. Coping mechanisms used by family members	Acceptance of MHCUs conditions Love and respect for MHCUs Effective continuous communication with MHCUs Prayer for coping Pastoral counselling enhance family members coping
3. Suggestions to improve family members coping mechanisms	More mental healthcare facilities are needed locally Families need intervention by the police.

MHCUs, mental health care users.

#### Theme 1: Challenges experienced by family members

Six sub-themes emerged from the challenges experienced by family members of MHCUs: difficult, uncooperative behaviour by MHCUS, MHCU substance use, defaulting treatment stigma because of mental illness, negative attitude from the nursing staff, insufficient income to care for MHCUs.

**Difficult uncooperative behaviour by mental health care users:** The majority of the participants reported that at times MHCUs present difficult and uncooperative behaviour. The participants mentioned that the MHCUs may refuse to bathe, eat, or be taken care of by other family members. Mental health care users may also refuse to go to health centres or hospitals or even refuse to take treatment. In concurrence with the study’s findings, participants expressed the following:

‘The main challenge at first was that he refused to bath, refused to go to the clinic or doctor.’ (Participant B, Female, 63 years old)

‘When I have to go somewhere, he refuses to be left with other people.’ (Participant A, Female, 57 years old)

‘Even our communication is not good because when you tell him something, he says the opposite.’ (Participant D, Female, 55 years old)

**Mental health care users substance use:** The participants expressed that some of their challenges and stress are a result of substance use by the MHCUs. Substance use results in aggressive behaviours which consequently stresses the family members. Most participants have articulated that:

‘We take them to (mentioned name of hospital) they give them treatment, but they don’t drink those pills even they admit them when they come back, they use drugs again.’ (Participant G, Female, 56 years old)

‘For an example when I have left food for us to eat them the following day, he eats them, and he refuse to quit cannabis.’ (Participant C, Female, 38 years old)

**Defaulting treatment:** Participants confirmed that defaulting treatment by the MHCUs is one of the challenges they are faced with. Examples included skipping medication and not adhering to their treatment. Below are some of the views participants shared:

‘We take them to (mentioned name of hospital), they give them treatment, but they don’t drink those pills even they admit them when they come back, they use drugs again.’ (Participant G, Female, 56 years old)

‘Ok, well so at times, my father skips drinking his medication we have to monitor him if he has taken his medication sometimes he says he took them while he did not take them.’ (Participant H, Female, 49 years old)

**Stigma because of mental illness:** Participants expressed that they are stigmatised by some of the community members who have negative opinions and attitudes towards MHCUs, and their family members. This stigma leads to ostracisation which results in feeling the need to keep MHCUs away from the community. One of the participants concurred:

‘Yes, sir there is also the problem of stigma in the community as other community members have accepted the mentally ill patient and others don’t accept her.’ (Participant A, Female, 57 years old)

The views shared by some of the other participants were:

‘The other thing is that other people in the community will greet him and laugh then he will become violent.’ (Participant C, Female, 38 years old)

‘The (mentioned name of the community) community members wanted to beat him up then the headman of the village stopped the community members and called me then I told him that he is mentally ill.’ (Participant G, Female, 56 years old)

**Negative attitude from the nursing staff:** Participants reported that they experience negative attitudes from the nursing staff and they reported episodes of getting ill-treated when they accompany the family members to hospital. Participants revealed:

‘Another challenge is that when my daughter has episode and I take her to the hospital, at the hospital they ill-treat us especially the nurses, they don’t treat us well at all.’ (Participant A, Female, 57 years old)

‘Sometimes nurses at hospital are impatient, shout at my child and I don’t like it.’ (Participant E, Female, 27 years old)

**Insufficient income to care for mental health care users:** Participants revealed that their income is insufficient to take care of MHCUs and their basic needs. Participants mentioned that their income does not cover other important things like transport to take them to the clinic or hospital for treatment. Participants comments included:

‘At home we live together only the two of us and he does not work when he has to come to the clinic at times he does not have transport money, and he will go around asking money for the taxi like today he is supposed to go to (mentioned name of the hospital) he does not have transport money.’ (Participant J, Female, 42 years old)

‘Sometimes it is difficult financially to take care of my father, especially when he has to go to the clinic for monthly treatment or to hospital when he is not well.’ (Participant H, Female, 49 years old)

#### Theme 2: Coping mechanism used by family members

The following five sub-themes emerged from the coping mechanisms used by family members: acceptance of MHCU’s conditions, love and respect for MHCUs, effective continuous communication with MHCUs, prayer for coping and pastoral counselling to relieve stress.

**Acceptance of mental health care users’ conditions:** Participants expressed that they felt that MHCUs’ conditions should be accepted and ought to be treated with care and support like normal human beings. One participant mentioned this:

‘My child like any other child is a gift from god and I accept her for how she is, and she did not choose to be mentally ill.’ (Participant A, Female, 57 years old)

Another participant added:

‘What can help us to cope firstly we have to accept our situations, because if you don’t accept your situation you will not cope and accept and love your child.’ (Participant I, Female, 36 years old)

**Love and respect for mental health care users:** Participants expressed that MHCUs need to be loved and respected and that loving and respecting MHCUs improves their recovery which in turn reduces participants stress levels. Participants expressed:

‘Just treat him like a normal person like when I am not home my children love and respect him because he is their uncle, they don’t treat him like he is crazy.’ (Participant C, Female, 38 years old)

‘Our relatives have to love their children and be patient with them don’t be too harsh on them just reprimand them like any other child, because even if they are mentally ill, they can see if you love them and treat them like your other children.’ (Participant D, Female, 55 years old)

**Effective continuous communication with mental health care users:** Participants expressed that effective continuous communication with MHCUs might greatly reduce their symptoms and improve chances of recovery, and in turn reduce participants’ stress. Participants articulated:

‘Sometimes nurses communicate with us continuously, for example, they would call me to remind me to come collect his treatment and they also give me information about his illness.’ (Participant F, Female, 50 years old)

‘One day professionals invite a group of parents or people who take care of mental ill patients and motivate us telling us about the advantages and disadvantages, what can we do and tell us if there is a cure for mental illness or our children will suffer from it till death we must accept them as they are.’ (Participant E, Female, 27 years old)

**Families use prayer for coping:** Some participants indicated their beliefs in prayer as a coping mechanism because it helped them to manage their challenges and stress. Participants shared:

‘I don’t want to lie my only coping mechanism is a prayer I pray every day before I sleep asking the lord to help me.’ (Participant G, Female, 56 years old)

‘We just pray because he likes to pray, I travel a lot with work so when I am not home my children will pray, he likes it when we pray that someone put their hands on him then he will sleep after.’ (Participant C, Female, 38 years old)

**Pastoral counselling to relieve stress:** Some participants explained that pastoral counselling was effective in helping them cope with stress and that it gave them strength to continue providing care and support to MHCUs. The participants vocalised:

‘I am a Christian and I love praying. So praying and my pastor’s counselling helps me a lot as I have accepted my daughter’s situation because of the counselling from the pastor.’ (Participant A, Female, 57 years old)

‘It is only prayer and talking to my pastor, I don’t have any other way.’ (Participant B, Female, 63 years old)

#### Theme 3: Suggestions to improve coping

Sub-themes that emerged from suggestions to improve coping include more mental healthcare facilities and intervention by the police.

**More mental health care facilities are needed locally:** Participants expressed that there is a need for more local mental healthcare facilities in the local sub-district. They indicated that these facilities will increase access to mental healthcare and assist family members to cope effectively. Participants explained:

‘I think if government can build more psychiatric clinics or hospitals nearby for example I was living in (mentioned where she is staying) thinking I am nearer to (mentioned name of clinic), because when he was giving me problems I will call his brother to take us to the clinic so I think when there are clinics near and we have a problem it will be easy to get help fast.’ (Participant B, Female, 63 years old)

‘At … they keep children like this they keep them and then they are given activities sort of rehabilitating them. I was wondering if the government can take these kids to rehab because these children are many if you can go to (mentioned name of hospital) you will be hurt.’ (Participant G, Female, 56 years old)

**Families need timely interventions by the police:** Participants explained that timely intervention by the police during mental healthcare emergencies would be of great help to enable them to cope with their situation. Participants said:

‘The police are very helpful, for example, when she was missing, we went to the police station to report her missing, when we arrived to the police station they told us there is a lady who was found wondering around, so we need their interventions at all the times ….’ (Participant F, Female, 50 years old)

‘I think the police will help by talking to him because when he sees the family members he does not listen but when police talks he listen, I think the police can assist us to cope.’ (Participant B, Female, 63 years old)

## Discussion

The aim of the study was to explore, describe and contextualise coping mechanisms used by the families of the MHCUs in Mahikeng sub-district, NWP, South Africa. Additionally, the study aimed to make recommendations for improving coping mechanisms of the families of the MHCUs. The study revealed three themes namely; challenges experienced by family members, coping mechanism used by family members, and suggestions to improve family members coping mechanisms.

### Challenges experienced by family members

Challenges experienced by family members emerged as the first theme of this study. Sub-themes under this theme were that MHCUs at times demonstrated difficult and uncooperative behaviours such as refusing to bathe, eat, or taken care of by other family members. Kito and Suzuki (2016:375), contend that cooperating with family members particularly on maintaining personal hygiene and physical care gives MHCUs a sense of being clean and helps build a bridge across the divide of mind and body. In addition, these behaviours were exacerbated by MHCUs’ substance use which at times resulted in aggressive behaviour. Substance use is a major challenge in the management of MHCUs. Blanco et al. (2016:389), indicate that the use of substances such as cannabis or other substances by MHCUs increases the risk of depression, anxiety, aggressive behaviour and often leads to defaulting of treatment. Furthermore, it becomes increasingly difficult for family members to deal with or manage MHCUs when they are under the influence of substances (Blanco et al. 2016:389). The issue of defaulting on psychiatric medication was also reported by family members as a critical challenge (Buchman-Wildbaum et al. 2020:1). Defaulting treatment is known to have detrimental consequences for both MHCUs and their family members. These include increased symptom severity, relapses, re-hospitalisations and reduced quality of life (Buchman-Wildbaum et al. 2020:1).

A further challenge that was mentioned was stigmatisation experienced from community members. Experiencing stigma is severely damaging to MHCUs and their families, and may result in low self-esteem, feeling of shame, need to isolate the MHCUs from the community, high levels of stress and overall poor quality of life (Valery & Prouteau 2020:1–2). Participants further reported that they often experience negative attitudes from the nursing staff and at times were ill-treated when they accompanied the MHCUs to hospital. Keuroghlian et al. (2016:568) concurred with this and stated that the attitudes of nursing staff towards MHCUs have been observed to be negative, to have a higher degree of anger, irritation, hostility, with less liking, empathy, and nurturance. Furthermore, such negative attitudes towards MHCUs lowers the quality of care they receive and may worsen their clinical outcomes (Keuroghlian et al. 2016:568).

Another major challenge which emerged was the financial impact of taking care of MHCUs. Rohanachandra, Amarabandu and Rohanachandra (2020:1) add that caring and supporting MHCUs has implications for family members including socio-economic disadvantages, such as spending money in caring for MHCUs. Participants reported that their income was often insufficient to take care of MHCUs and their basic needs.. The inability to manage these costs may lead to stress for family members and poor quality care for MHCUs (Rohanachandra et al. 2020:1).

### Coping mechanisms

Different coping mechanism used by family members emerged as the second theme of the study. This includes acceptance of the condition, respect and loving the MHCUs and access to spiritual support. Acceptance of the MHCUs’ condition was the first sub-theme that emerged in this theme. According to Aldersey and Whitley (2015:468), family members’ involvement, acceptance and support of MHCUs, reduce the stress levels of the families and increase resilience, which enables them to cope effectively. Accepting MHCUs as family members increases the motivation to continue to care for and support them (Aldersey & Whitley 2015:468). Dindo, Van Liew and Arch (2017:548) further argue that family members should accept MHCUs and make efforts, particularly those consistent with supporting and caring for them. Moreover, family members should shield MHCUs from stigma and discrimination. Family members should help MHCUs with treatment adherence and compliance, and teach people to accept and treat MHCUs as normal human beings (Dindo et al. 2017:548).

Together with acceptance, a sub-theme emerged around the need for the MHCUs to be loved and respected. Loving and respecting MHCUs improves their recovery and in turn reduces participants stress levels (Iseselo, Kajula & Yahya-Malima 2016:2). Furthermore, social support is important for the well-being of the family members of MHCUs. Musyimi et al. (2016:2) also support the fact that a positive attitude, love and respect towards MHCUs can significantly reduce their symptoms. When MHCUs are accepted, and treated with respect, this improves their recovery and in turn reduces family members’ stress (Iseselo et al. 2016:2). Building on acceptance, love and respect was the need for effective continuous communication with MHCUs. Grover and Pradyumna (2015:7) reported that positive and effective communication with MHCUs positively affects their symptoms and recovery. Communicating could include information about the MHCUs’ condition, treatment and encouragement expressions on how they feel. In addition, it is important for family members to communicate with MHCUs about treatment side effects and gain their cooperation (Grover & Pradyumna 2015:7). McCabe et al. (2016:517) stated that improving communication is central to improving the relationship between family members and MHCUs. They further argue that a lack of shared understanding between family members and MHCUs can lead to more disagreements.

The role of prayer and pastoral support in coping were two further subthemes. Most of the participants mentioned that they use prayer as a coping mechanism. Fife, Brooks-Cleator and Lewis (2020:4) state that a prayer is a specific coping mechanism that is commonly used by family members of MHCUs who believe that God will help them in their challenges. Furthermore, reading the Bible has been identified as a coping mechanism that also inhibits the negative impacts of stress on an individual’s hope for the future (Fife et al. 2020:4). Von Kardorff et al. (2016:6) confirm that some of the coping mechanisms used by family members of MHCUs include use of prayers. In addition, pastors and other divine instructors may offer such prayers hoping for miracles as a way of coping and dealing with the stress from caring for MHCUs (Von Kardorff et al. 2016:6). Participants explained that pastoral counselling is helping them to cope with their stress and that it gives them strength to continue providing care and support to MHCUs. A relationship with God, gives family members meaning and purpose beyond themselves. Gonçalves et al. (2015:2938) add that prayer and pastoral counselling as a coping mechanism for family members has a direct relationship with their psychological well-being, such as satisfaction, happiness and moral values. Family members who engage in religious activities such as prayers and pastoral counselling reported that they were often less stressed, had increased motivation to care for MHCUs, were content, filled with purpose, their lives had meaning, and they had a positive outlook towards life (Gonçalves et al. 2015:2938).

### Suggestions to improve families abilities to cope with caring for a mental health care users

The last theme that emerged from the findings of this study was suggestions to improve family mechanisms for coping with MHCUs. The first sub-theme was around access to mental healthcare treatment facilities. Participants revealed that there is a need for more local mental healthcare facilities in their sub-district. Torrey et al. (2015:5), explain that there is a need for more local public mental healthcare institutions for MHCUs in order for them to access free mental healthcare. Ohtani et al. (2015:798), argue that for MHCUs to gain access to mental healthcare, there should be more local mental healthcare institutions in which the language spoken by the healthcare providers should be understandable to the MHCUs and their family members.

A second sub-theme was around the timeous intervention by the police when MHCUs are difficult to handle. Omoaregba et al. (2015:57) state that the police are often the first community resource called upon to respond to urgent situations involving the mental healthcare emergency. For example, when a MHCU is violent towards their family members, the police would ensure that the family members are protected from harm and that the MHCU is transported safely to a psychiatric hospital for treatment (Omoaregba et al. 2015:57). Livingston (2016:850), add that police officers attend to mental health emergencies when there is violence between MHCUs and family members or the community members. In such situations, the police ensures that they restore order and protect those who are at risk of harm, whether it’s the MHCUs or not; and where necessary the police officers always ensure that the MHCU receives mental healthcare assistance.

### Limitations

The study was conducted in three community health centres in Mahikeng sub-district in NWP of South Africa and cannot be generalised to other community health centres in the province or in the country.

### Recommendations

The study recommends further research on the strategies to improve coping mechanism of the families of the MHCUs. The study adds to what is already known about the topic and also recommends that more research be conducted regarding more coping mechanisms used by the families of MHCUs in order to improve them and effectively improve their well-being.

## Conclusion

The findings of this study highlighted that the participants were faced with diverse challenges. Their challenges include unruly behaviour fuelled by substance use by MHCUs which often results in MHCUs defaulting treatment. In addition, the participants experienced stigma from the community and negative attitudes from the nursing staff. This was compounded by the financial strain in caring for a MHCU and the need for increased access to mental healthcare and timely support from police. A positive outcome of the study was the coping mechanisms currently used by family members including acceptance of MHCUs conditions, love and respect for MHCUs, effective continuous communication with MHCUs, use of prayer for coping and pastoral counselling.
